# Herpesviruses control the DNA damage response through TIP60

**DOI:** 10.1186/1750-9378-7-S1-O8

**Published:** 2012-04-19

**Authors:** Renfeng Li, Jian Zhu, Zhi Xie, Gangling Liao, Shaohui Hu, Crystal Woodard, Jimmy Lin, Gary S Hayward, Jiang Qian, Heng Zhu, S Diane Hayward

**Affiliations:** 1 Department of Oncology, Johns Hopkins School of Medicine, Baltimore, MD, USA; 2 Department of Pharmacology and Molecular Sciences, Johns Hopkins School of Medicine, Baltimore, MD, USA; 3 High Throughput Biology Center, Johns Hopkins School of Medicine, Baltimore, MD, USA; 4 Department of Ophthalmology, Johns Hopkins School of Medicine, Baltimore, MD, USA; 5 Kimmel Cancer Center, Johns Hopkins School of Medicine, Baltimore, MD, USA

## Background and results

**Figure 1:**
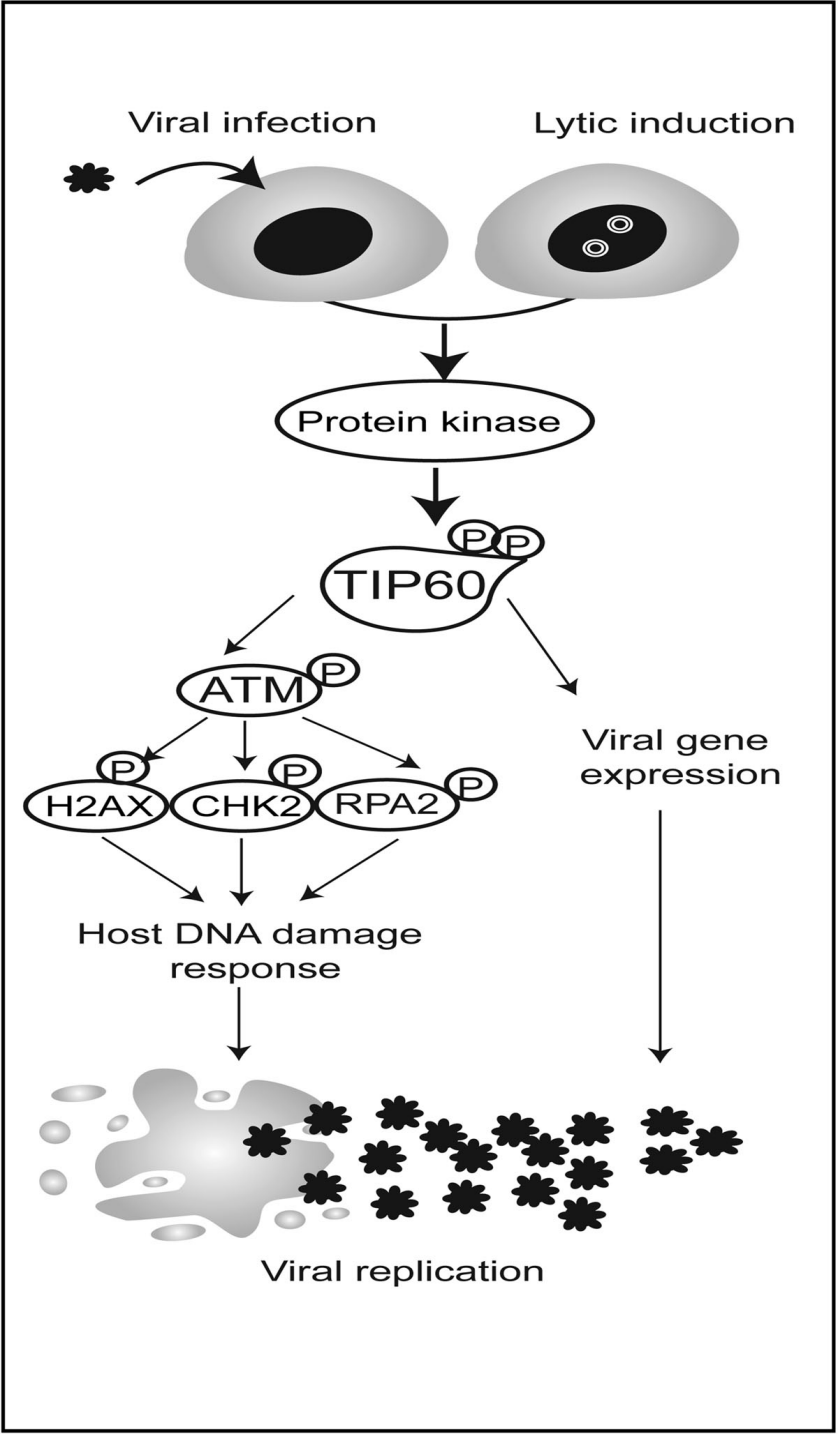


Herpesviruses establish life-long persistent infections that result in clinical manifestations ranging from mild cold sores, to pneumonitis and cancers. Immunosuppressed populations, including AIDS patients, are at risk for more serious disease outcomes. Although the α-, β-, and γ-herpesviruses infect different tissues and cause distinct diseases, they confront many of the same challenges in producing new virions and spreading infection. The herpesvirus families each encode a conserved serine/threonine kinase that plays an important role in virus replication and spread. Despite the potential of these kinases as pharmacological targets, the extent of substrate conservation and the key common cell signalling pathways targeted by these enzymes are unknown. We applied a human protein microarray, high-throughput approach to identifying shared substrates of the conserved kinases from herpes simplex virus, human cytomegalovirus, Epstein-Barr virus (EBV) and Kaposi’s sarcoma associated herpesvirus. We identified 110 shared host substrates targeted by at least three conserved viral kinases. Bioinformatics analyses revealed that proteins involved in the DNA damage response (DDR) were statistically enriched and further orthogonal analysis led to an in-depth characterization of a histone acetyltransferase, TIP60, as a master regulator that is exploited by these viruses. In EBV replication, TIP60 acts both by triggering the EBV-induced DDR and by regulating expression of viral lytic genes (Figure 1).

## Conclusions

1. The conserved herpesvirus kinases target the DNA damage response (DDR) pathway.

2. The EBV kinase BGLF4 induces the DDR and regulates key lytic viral genes through TIP60.

3. TIP60 knockdown impairs and γ herpesvirus replication.

4. Identification of key cellular targets of the conserved herpesvirus kinases will facilitate the development of broadly effective anti-viral strategies.

